# Reduction in Pathogenic Biofilms by the Photoactive Composite of Bacterial Cellulose and Nanochitosan Dots under Blue and Green Light

**DOI:** 10.3390/jfb15030072

**Published:** 2024-03-14

**Authors:** Danica Z. Zmejkoski, Nemanja M. Zdravković, Milica D. Budimir Filimonović, Vladimir B. Pavlović, Svetlana V. Butulija, Dušan D. Milivojević, Zoran M. Marković, Biljana M. Todorović Marković

**Affiliations:** 1 Vinča Institute of Nuclear Sciences, National Institute of the Republic of Serbia, University of Belgrade, 11001 Belgrade, Serbia; mickbudimir@gmail.com (M.D.B.F.); svetlana8@vin.bg.ac.rs (S.V.B.); dusanm@vinca.rs (D.D.M.); zoranmarkovic@vinca.rs (Z.M.M.); biljatod@vinca.rs (B.M.T.M.); 2 Scientific Institute for Veterinary Medicine of Serbia, Janisa Janulisa 14, 11107 Belgrade, Serbia; nemanja.zdravkovich@gmail.com; 3 Faculty of Agriculture, University of Belgrade, Nemanjina 6, 11080 Belgrade, Serbia; vlaver@agrif.bg.ac.rs

**Keywords:** chitosan nanoparticles, bacterial cellulose, photoactive therapy, antibiofilm, nanocomposite hydrogels, blue light and green light

## Abstract

In this study, nanochitosan dots (ChiDs) were synthesized using gamma rays and encapsulated in bacterial cellulose (BC) polymer matrix for antibiofilm potential in photodynamic therapy. The composites were analyzed for structural changes using SEM, AFM, FTIR, XRD, EPR, and porosity measurements. Additionally, ChiD release was assessed. The results showed that the chemical composition remained unaltered, but ChiD agglomerates embedded in BC changed shape (1.5–2.5 µm). Bacterial cellulose fibers became deformed and interconnected, with increased surface roughness and porosity and decreased crystallinity. No singlet oxygen formation was observed, and the total amount of released ChiD was up to 16.10%. Antibiofilm activity was higher under green light, with reductions ranging from 48 to 57% under blue light and 78 to 85% under green light. Methicillin-resistant *Staphylococcus aureus* was the most sensitive strain. The new photoactive composite hydrogels show promising potential for combating biofilm-related infections.

## 1. Introduction

The large-scale use of antibiotics (in medicine, veterinary medicine, agriculture, etc.) has led to the development of antibiotic resistance in bacteria. The biofilm formed by pathogenic bacteria, consisting of extracellular polysaccharides, extracellular DNA, and proteins, acts as a barrier for many antibiofilm compounds and is considered an important resistance mechanism against modern antibiotics [1,2]. They are commonly associated with medical devices and tissue-related conditions, playing a crucial role in reducing antimicrobial efficacy and immune responses, thereby leading to persistent and chronic infections. There is extensive drug resistance in bacteria, even leading to the development of multi-drug-resistant bacteria [3], whose expansion has become a major challenge in the development of new antibiotic substitutes [4]. Furthermore, the possibility of the formation of a bacterial biofilm complicates the medical state of the patients, increasing bacterial tolerance to antimicrobials, directing evolutionary pressure toward resistant microorganism strains, and facilitating gene transfer [5], resulting in a significant increase in mortality and morbidity in patients with wounds [6,7]. Polymicrobial biofilms, in particular, pose a formidable challenge, making treatment difficult or impossible. Understanding the composition and mechanisms of the action of biofilms is essential across various fields, including infection and transmission dynamics, biofouling, and bioenergy. Increasing antibiotic doses within maximal therapy levels may not result in antibiofilm effects, and simultaneously, the biofilm significantly reduces the ability of immune system components to reach the infection site [8]. In addition, biofilms on abiotic surfaces lead to severe foodborne and nosocomial infections [9,10].

In bacteria, conventional antibiotic resistance mechanisms like point mutations, enzymes, and efflux pumps are often ineffective against biofilm organisms. Instead, various components within a biofilm cooperate to diminish or completely thwart antibiotic efficacy, perpetuating resistance. These combined mechanisms enable organisms to survive within the biofilm despite exposure to high antibiotic concentrations, which is a phenomenon known as recalcitrance.

Greater awareness of the persistent threat posed by biofilms in the hospital setting and their role in facilitating the exchange of resistance mechanisms is essential. As antimicrobial stewardship and infection prevention programs advance, it becomes increasingly crucial to comprehend the risks associated with biofilms. Preventing the transfer and acquisition of biofilm-causing organisms can significantly impact the spread of antimicrobial resistance.

Among biofilm-forming bacteria, the most infamous are *Staphylococcus aureus*, methicillin-resistant *Staphylococcus aureus* (*MRSA*), *Pseudomonas aeruginosa*, and *Escherichia coli* and due to their resistance to antibiotics and the immune system, they pose a global threat to public health [11]. In particular, *MRSA* is among the antibiotic-resistant bacteria posing the greatest threat to human health [12]. Nanoplatforms with antimicrobial properties linked to photodynamic and photothermal therapies present promising options for tackling antimicrobial resistance. These alternative treatments offer non-invasive, antibiotic-free solutions with dual selectivity and minimal adverse effects in therapeutic procedures [13]. Antimicrobial photodynamic therapy could be an ideal technique for the obliteration of multi-drug-resistant microorganisms [14,15]. A light source and light-sensitizing agents (photosensitizers, PSs) are widely used in photodynamic therapy intended to treat various infections caused by bacteria, fungi, and viruses [16]. Via the absorption of visible light, PSs are activated to form the excited singlet state. Afterward, they transition to a long-lived excited triplet state, which, in the presence of oxygen, forms reactive oxygen species that destroy pathogenic agents. Singlet oxygen, as a very strong oxidant, is the main agent of photooxidative stress in microorganisms which oxidizes cellular lipids, proteins, DNA, and RNA, yet it has a high potential to diffuse across cellular membranes into extracellular compartments [17] like the extracellular components of biofilms actually are.

Antimicrobial photodynamic therapy has distinct advantages over antibiotics. Firstly, it exhibits triple site-specificity, minimizing systemic toxicity by preferentially targeting infected cells while sparing non-target cells. Unlike antibiotics, this kind of therapy does not induce resistance even after repeated treatments due to its short drug–light interval and lack of dark toxicity [18]. Additionally, bacteria struggle to detect the oxidative stress caused by PDT, hindering their ability to adapt metabolically [19]. Moreover, photodynamic therapy damages multiple bacterial sites, making it effective against resistant strains and offering a minimally invasive treatment option.

Chitosan is a non-toxic, biodegradable, cost-effective, biocompatible, and natural polymer [20] consisting of partially alkaline-deacetylated chitin arising from the following two monosaccharides: glucosamine and N-acetyl glucosamine. The nanoparticle forms of chitosan demonstrate stability at high temperatures and are resistant to microbial and enzymatic degradation [21]. They are effective in neutral pH environments while retaining positively charged amino groups [22]. This positively charged characteristic enables chitosan to interact with negatively charged residues on microbial cytoplasmic membranes (lipids, carbohydrates, and proteins), thereby blocking membrane permeability and leading to the leakage of the cytoplasmic content, which is the proposed mechanism underlying its antibacterial activity [23]. In contrast to bacterial prokaryotic cell morphology, eukaryotic cells have a very different organization; therefore, chitosan and chitosan-based polymers and nanoparticles are proven to be non-toxic for human tissues [20,24]. Furthermore, they are favorable in wound healing [25] and even in treating arthritis [26]. There are promising results in chitosan-based materials intended for use in sites with a heavy bacterial burden, such as the oral cavity [27], even for orthodontic composites [28]. To date, only chitosan-based drug delivery systems have been investigated in photodynamic therapy [29,30,31].

The objective of this study was to assess the antibiofilm efficacy of nanochitosan dots (ChiDs) within a hydrogel-based composite containing bacterial cellulose when subjected to blue and green light exposure. This investigation was prompted by prior research indicating the photoactivity of ChiDs [25]. In brief, ChiDs emit green luminescent light and have photoluminescence (PL) at an excitation-emission of 530 nm. Their encapsulation in bacterial cellulose does not change their PL emission, and their composite shows the highest PL emission at a 480 nm excitation wavelength. Blue and green light, as a range of the visible light spectrum, have a wavelength between 400 and 525 nm and 500 and 600 nm, respectively. Since blue and green light do not penetrate the skin more than 2.5 mm, their application in photodynamic therapy is limited to open surfaces [32]. The hydrogel as a carrier of the antimicrobial agent was chosen as one of the ideal materials for topical use [33]. In our previous studies, bacterial cellulose showed good properties in wound treatment [34,35]. Also, bacterial cellulose has potential as a coating in many fields [36,37,38].

In order to measure the antibacterial effect by means of bacterial cell abundance inside biofilms, a tetrazolium salt-based MTT ((3-(4,5-dimethylthiazol-2-yl)-2,5-diphenyltetrazolium bromide) assay was developed [39]. Due to bacterial cell respiratory chain processes, the reduction in MTT to purple formazan occurs, which can be measured by spectrophotometry. The intensity of the change is proportional to live bacteria cells; thus, this is called the viability assay, allowing the measurement of quantities of live cells in biofilm [40].

In this study, composite hydrogels of bacterial cellulose and ChiDs were proposed as photoactive agents in *in vitro* antibiofilm eradication. Since these hydrogels previously showed a very good antibiofilm effect in ambient light conditions, exposure to blue and green light might improve this effect via morphological changes or singlet oxygen formation.

## 2. Materials and Methods

### 2.1. Synthesis of Nanochitosan Dots (ChiDs), Bacterial Cellulose (BC) and Preparing Photoactive Composites BC-ChiD

Nanochitosan dots (ChiDs) were obtained by the following previously described approach: the gamma irradiation of low-molecular-weight Chi (50–190 kDa, (Merck KGaA, Darmstadt, Germany) solutions at 60 kGy [25]. Bacterial cellulose (BC) was provided by the Institute of Molecular Biology and Genetics, Kyiv, Ukraine, sourced from the bacterial culture *Komagataeibacter intermedius* IMBG180 (part of the microorganism collection of the Institute of Molecular Biology and Genetics, Kyiv, Ukraine). The composites, designated as BC-ChiD, were prepared by immersing BC into 0.2% and 2% ChiD solutions for 48 h. The composite samples exposed to blue light (470 nm, 3 W, 30 min) and green light (537 nm, 3 W, 30 min) were labeled as BC-ChiD_blue and BC-ChiD_green, respectively. The light source was at a distance of 20 cm, and no temperature change near the samples or plates was registered. The composite samples exposed to ambient light were used as a control (BC-ChiD_control). All samples were lightly blotted on filter paper to remove excess solution before being subjected to physiochemical and biological characterization. The samples immersed in 2% ChiD solutions showed better-recognized spectra, peaks, and surface morphology than composite hydrogels with 0.2% ChiDs; therefore, they were used for physiochemical methods.

### 2.2. Characterization Methods

#### 2.2.1. Scanning Electron Microscopy (SEM) Imaging

The surface morphology of air-dried BC-ChiD_control, BC-ChiD_blue, and BC-ChiD_green composite samples was analyzed by scanning electron microscopy (SEM) using the JEOL JSM-6390LV microscope (Jeol USA Inc., Peabody, MA, USA), conducted in a vacuum at room temperature with an accelerating voltage of 25 kV.

#### 2.2.2. Fourier Transform Infrared (FTIR) Absorption Spectroscopy

In order to assess the structural differences between BC-ChiD_control and BC-ChiD composites exposed to blue and green light, samples were air-dried, and FTIR measurements were conducted using an infrared microscope, Nicolet iN10 Thermofisher Scientific (Thermo Electron Scientific Instruments LLC, Madison, WI, USA) operated in the ATR mode. All measurements were conducted in the air at ambient temperatures in the range of 400 to 4000 cm^−1^, with a spectral resolution of 4 cm^−1^.

#### 2.2.3. X-ray Diffraction (XRD)

The XRD measurements of BC-ChiD_green and BC-ChiD_blue samples were performed using Rigaku Ultima IV (Tokyo, Japan). The X-ray beam was nickel-filtered CuKα1 radiation (λ = 0.1540 nm, operating at 40 kV and 40 mA). The XRD data were scanned at an angle 2θ between 3° and 90°. To deconvolute the specific region of crystal/amorphous peaks, we used Origin Lab software (Origin 8.5, OriginLab Corporation, Nrthampton, MA, USA) based on the assumption of the Gaussian function. The percentage of crystallinity was determined using the following equation:

(1) $$X_{C}=\frac{A_{C}}{A_{T}}\times 100\%$$

where *X_C_* is the degree of crystalline in the percentage, *A_C_* is an area of the crystalline region, and *A_T_* is the total region under the peak (including crystalline *A_C_* and amorphous *A_a_* area) [41].

Using Scherrer’s equation, the crystallite size of the sample was calculated from the XRD pattern:

(2) $$D=\frac{K\lambda }{\beta \cos\theta }$$

where *λ* is the X-ray wavelength, *β* is the full width at half maximum (FWHM), *θ* is the Bragg angle for the studied peak, and *K* is the shape factor.

#### 2.2.4. Atomic Force Microscopy (AFM) Imaging

Due to its high-resolution topography, ability to work under ambient conditions, and capability to reconstruct 3D images, AFM has proven to be a very powerful technique for imaging microbial surfaces [42,43,44]. Pathogenic bacteria biofilms treated with BC-ChiD_control and BC-ChiD composite samples under blue and green light were fixed (hot air drying) and analyzed using an AFM Quesant microscope (Ambios Technology, Santa Cruz, CA, USA) operating in the tapping mode. The AFM measurements were performed in the air using a silicone T-shaped cantilever with a spring constant of 40 N/m. All images were obtained at 2 Hz with a 512 × 512 image resolution over different square areas. Detailed information on the surface topography of all samples was calculated using Gwyddion software (version 2.61, Czech Metrology Institute, Brno, Czech) [45]. The average surface roughness and surface area were calculated from several images of 10 × 10 µm^2^ in square size for each sample and were presented as values ± standard deviations.

#### 2.2.5. Porosity of BC-ChiD_Control, BC-ChiD_Blue and BC-ChiD_Green Composite Hydrogels

The liquid displacement method was used to determine the porosity of control and exposed composites [46]. Disc-shaped BC-ChiD samples (11 mm in diameter and 1 mm in height) were weighed (*W*1) and immersed in ethanol until saturation. The porosity of composite hydrogels was calculated by measuring the weight of samples after immersion (*W*2) using the following equation:

(3) $$P=\frac{W2-W1}{\rho \;V1}$$

where *V*1 is the volume of the sample and *ρ* is the density of ethanol [47]. The measurements were conducted in triplicate, and values were statistically analyzed using Student’s *t*-test.

#### 2.2.6. Electron Paramagnetic Resonance (EPR) Measurements of BC-ChiD_Blue and BC-ChiD_Green Composites

For EPR measurements, a Spectrometer MiniScope 300, Magnettech, Berlin, Germany, was used. The microwave power was 3.2 mW with a modulation amplitude of 0.2 mT. The instrument was operating at a nominal frequency of 9.5 GHz. 2,2,6,6-tetramethylpiperidine (TEMP) was used as a spin trap to detect singlet oxygen formation (^1^O_2_). The BC-ChiD_blue and BC-ChiD_green composites were dipped in a 30 mM TEMP solution in ethanol and were irradiated by blue light at 470 nm and green light at 532 nm, respectively, for up to 23 h in the closed and reflective chamber, together with control samples without BC-ChiD for intensity comparison.

#### 2.2.7. *In Vitro* Release of ChiD from BC-ChiD_Blue and BC-ChiD_Green Composites

The BC-ChiD composite samples were shaped as 22 mm diameter discs with an average thickness of 1 mm. The *in vitro* release of ChiD from BC-ChiD_blue and BC-ChiD_green composites was monitored in 15 mL PBS (pH 7.4) at 37 °C with constant shaking at 100 rpm. Aliquots of the sample (1 mL) were collected at predetermined time intervals (1, 2, 3, 4, 5, 6, 24, 48, and 72 h), and the released ChiD (as a percentage) in the dissolution media was determined spectrophotometrically using a Shimadzu spectrophotometer (SHIMADZU CORPORATION, Kyoto, Japan) at 290 nm. After measuring absorbance, the aliquots were immediately returned to the dissolution media to maintain a constant volume. All experiments were performed in duplicate.

#### 2.2.8. Photo-Induced Antibiofilm *In Vitro* Test

The antibiofilm activity was tested against a set of Gram-positive bacteria: *Staphylococcus aureus* (ATCC 25923), methicillin-resistant *Staphylococcus aureus* (*MRSA* ATCC 43300), as well as Gram–negative bacteria *Escherichia coli* (ATCC 25922), *Klebsiella pneumoniae* (ATCC 700603,) and *Pseudomonas aeruginosa* (ATCC 27853).

- Biofilm formation and plate preparation

Biofilm was formed in 96-well polystyrene titer plates. Bacterial inoculum was prepared from overnight culture suspension according to ISO 20776-1:2006 Clinical laboratory testing and *in vitro* diagnostic test systems—Susceptibility testing of infectious agents and evaluation of performance of antimicrobial susceptibility test devices—Part 1: Reference method for testing the *in vitro* activity of antimicrobial agents against rapidly growing aerobic bacteria involved in infectious diseases. International Organization for Standardization: Geneva, Switzerland, 2006 [48], finally containing 5 × 10^5^ CFU/mL. A sterile flat bottom polystyrene 96-well microtiter plate (Sarstedt, Nümbrecht, Germany) was filled with 100 µL CAMHB (BBL, Franklin Lakes, NJ, USA), and 5 µL of bacterial inoculum was incubated at 37 °C aerobically overnight. The supernatant was removed from each well, and the plates were rinsed three times using 100 μL of sterile physiological saline (PS). Subsequently, fresh medium in a volume of 100 μL was added to each well.

- Antibiofilm test

The composite materials formed in a disc shape (6 mm in diameter, 1 mm thickness) were immersed in the prepared wells. At this point, plates were exposed to green and blue light (λ = 537 nm, 3 W, BC-ChiDs_green; λ = 470 nm, 3 W, BC-ChiDs_blue) for 30 min (at a height of 20 cm) to initiate a potential photoactive effect, and plates exposed to ambient light were used as a control and blanks. The light cannot pass through the composites, and the dimensions of the samples are such that they prevent light from directly acting on the biofilms. After illumination, plates were reincubated at 37 °C aerobically overnight. After incubation, the samples and medium were removed, and each well in the plates was rinsed three times using 100 μL PS. A 3% solution of 3-(4,5-dimethylthiazol-2-yl)-2,5-diphenyltetrazolium bromide (MTT, Sigma) in CAMHB at a volume of 100 µL was added to each well. The plates were incubated at 37 °C aerobically for 2 h. Formazan crystals were dissolved in 100 µL DMSO (Zorka, Šabac, Serbia), and the absorbance was determined at 450 nm with a microplate reader. For negative controls (blanks), raw wells with material were concealed from green or blue light treatment. Wells with bacterial biofilm without materials but within light treatment were used to assess a possible light effect bias, with experiment rejection criteria if over a 5% average OD difference under different light regimes.

The experiment was conducted in duplicates, and the average absorbance was calculated. The results are presented as percentages. The percentage of biofilm reduction (BR) was calculated according to the formula [49]:

(4) $$BR=\frac{OD(blank)\;-\;OD(treatment)}{OD(blank)}\times 100$$

## 3. Results and Discussion

### 3.1. Surface Morphology of BC-ChiD_Blue and BC-ChiD_Green Composites

**Figure 1:**
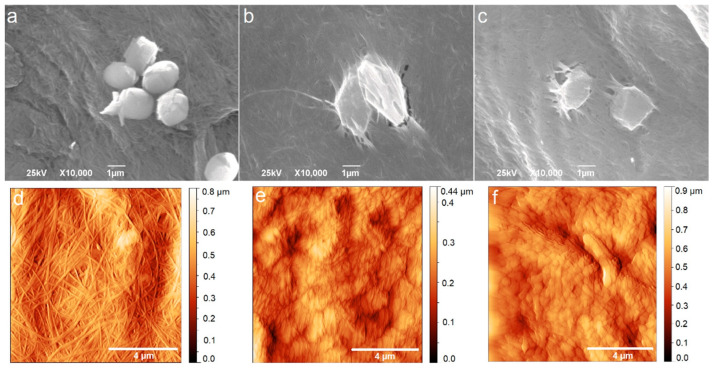
Scanning electron microscopy (SEM) and atomic force microscopy (AFM) images of BC-ChiD_control (**a**,**d**); BC-ChiD_blue (**b**,**e**); and BC-ChiD_green (**c**,**f**) samples.

**Figure 2:**
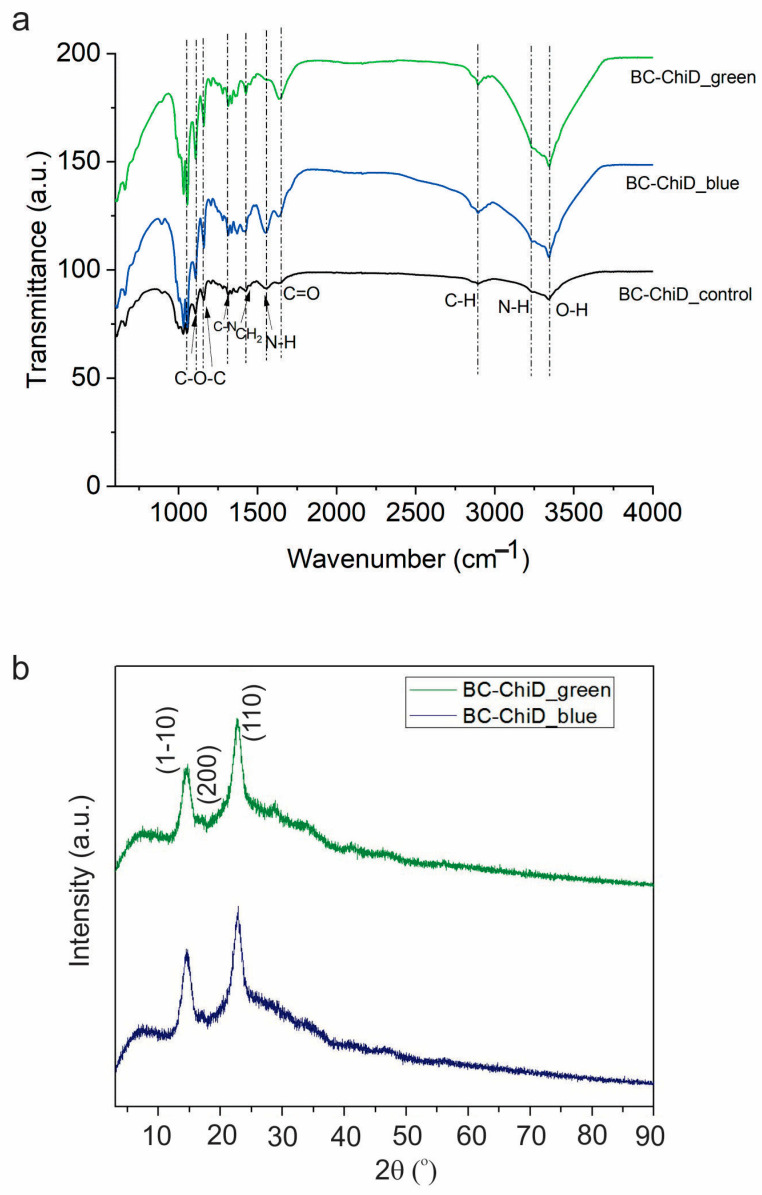
FTIR spectra (**a**) of BC-ChiD_control (black curve), BC-ChiD_blue (blue curve), and BC-ChiD_green (green curve). All spectra are displaced for clarity. XRD patterns (**b**) of BC-ChiD_blue and BC-ChiD_green samples.

Figure 1 and Figure 2 show the surface morphology of BC-ChiD samples under the following different conditions: ambient, blue, and green irradiation for 30 min. The light source used (LED, power 3 W) was neither coherent nor polarized, but it was randomly oriented and incoherent. The irradiation was perpendicular to the samples studied, and the distance between the light source and the sample surface was 20 cm. From Figure 1b,c, one can observe the changes in the shape of ChiD agglomerates embedded in a polymer matrix. After exposure to blue and green light, their size was between 1.5 and 2.5 µm. Our previous study [25] reported ChiDs’ average diameter of 50 ± 1.5 nm and average height of 1.8 nm.

From both SEM (Figure 1a–c) and AFM images (Figure 1d–f), we can notice that blue and green light influence the morphology of the BC-ChiD samples. The control sample BC-ChiD (Figure 1a,d) has a pronounced fibrous structure consisting of long and thin fibers of average width (100 ± 10) nm and lengths in the order of magnitude of ten micrometers. After exposure to blue and green lights, the bacterial cellulose fibers became deformed and interconnected, and the surface appeared more wrinkled than fibrous (Figure 1b–e). The length of the fibers notably decreased to 1–3 μm, while their widths increased to average (168 ± 15) nm and (219 ± 16) nm after the exposure to the blue and green light, respectively. Both average surface roughness (RMS) values and the surface area decreased after exposure to blue and green lights due to the mentioned change in the morphology. The average surface roughness (RMS) of BC-ChiD_control, BC-ChiD_blue, and BC-ChiD_green composites were 94.57 ± 2.88 nm, 77.69 ± 10.09 nm and 74.4 ± 7.25 nm, respectively. The surface area of BC-ChiD_control, BC-ChiD_blue and BC-ChiD_green composites were 173.42 ± 3.19 µm^2^, 137.62 ± 6.23 µm^2^ and 124.87 ± 5.85 µm^2^, respectively.

### 3.2. Chemical Composition

Figure 2a presents FTIR spectra of BC-ChiD samples irradiated by ambient, blue, and green light. From this figure we can detect the following peaks: peaks at 3340 and 3245 cm^−1^ stem from O-H and N-H vibrations, whereas the peak at 2895 cm^−1^ belongs to C-H stretching vibrations; the peak at 1646 cm^−1^ could be assigned to the C=O stretching of amide I; the peak at 1563 cm^−1^ could stem from N-H vibrations and is down-shifted compared to Chi powder peak at 1597 cm^−1^, whereas the peak at 1436 cm^−1^ originates from CH_2_ vibrations [50]; the peak at 1326 cm^−1^ could be assigned to the C-N stretching of amide III, whereas the peak at 1158 cm^−1^ was identified as the asymmetric stretching of the C-O-C bridge. The peak at 1059 cm^−1^ stems from C-O stretching vibrations. In our previous studies, we depicted the FTIR spectrum of neat BC samples [25].

The obtained FTIR spectra indicate that the chemical composition of all investigated samples did not change upon irradiation by different wavelengths.

Figure 2b shows a comparative analysis of X-ray diffraction (XRD) spectra for samples BC-ChiD_blue and BC-ChiD_green. In both samples, three distinct peaks appear at approximately 14, 16, and 22.6 2θ, corresponding to the (1–10), (200), and (110) crystal planes of type I-β cellulose [51]. Additionally, a peak at ~28.8 2θ is evident in the XRD spectra, with a more pronounced presence in the BC-ChiD_green sample. This specific peak is attributed to ChiDs [52]. Notably, other ChiD-associated peaks at 11.7, 16.4, 18.4, and 22.3 2θ [49] are not discernible in the spectrum due to their positions overlapping with the higher-intensity signals from BC. The degree of crystallinity and crystallite size for each sample are detailed in Table 1.

In our previous research [25], it was demonstrated that the BC-ChiD_control exhibited a 74% degree of crystallinity. It is widely acknowledged that neat BC possesses an inherently high crystallinity index [53]. When synthesized, BC undergoes processing with various compounds, or when these compounds are introduced into the culture medium, there is a discernible alteration in the crystalline constituents of BC [54,55], resulting in a subsequent decrease in the degree of crystallinity [56,57]. This observation aligns with the findings of the present study.

The reduction in crystallinity, compared to the BC-ChiD_control, is consistent with the results obtained from scanning electron microscopy (SEM) and atomic force microscopy (AFM). Evidently, the light treatment exerts an influence on the morphology of BC fibers within the treated samples, thereby affecting their crystallinity.

### 3.3. Porosity of BC-ChiD_Control, BC-ChiD_Blue and BC-ChiD_Green Composite Hydrogels

**Figure 3:**
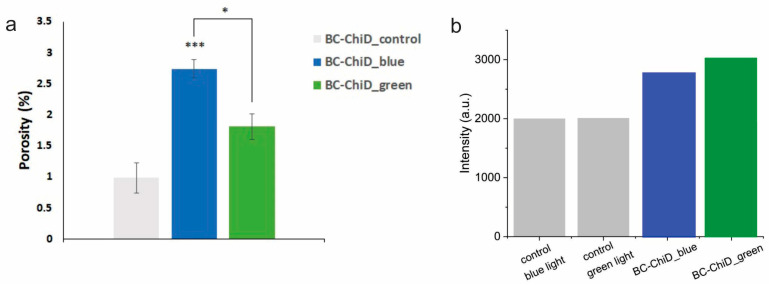
Porosity (**a**) of BC-ChiD_control, BC-ChiD_blue, and BC-ChiD_green composite hydrogels. (* *p* < 0.05; *** *p* < 0.001). EPR signal intensity (**b**) of control samples, as well as BC-ChiD_blue and BC-ChiD_green samples.

Since the porosity of materials represents one of the important parameters for their potential usage in medicine [58], the analyses of BC-ChiD_control, BC-ChiD_blue, and BC-ChiD_green composite hydrogels were conducted. As can be seen from the results of the BC-ChiD_control, BC-ChiD_blue, and BC-ChiD_green composite hydrogels porosity, as presented in Figure 3a, the values of composites exposed to blue light were significantly higher (*p* < 0.001) compared to the control and composites exposed to green light (*p* < 0.05). The results show that exposure to blue and green light significantly increases the pore dimension due to the specific bonds (NH_2_, C-N, C=O, and C-O-C) between BC fibers and ChiDs under different sources of light exposure.

### 3.4. EPR Measurement of Singlet Oxygen Formation

Figure 3b shows the TEMP spin trap EPR intensities of BC-ChiD samples illuminated with blue and green light. TEMP molecules rapidly react with ^1^O_2_, forming a stable, EPR-active product, TEMP-^1^O_2_ (TEMPO). Commercially available TEMP spin traps contain a small amount of TEMPO as an impurity, which contributes to a minor parasitic signal. However, upon exposure to singlet oxygen, the intensities of the spin trap EPR spectrum increase significantly, rendering the impurity signal negligible. The BC-ChiD and control samples were subjected to blue and green light, and no intensity differences higher than the experimental error were observed. Figure 3b shows intensities after one hour, but the intensities were followed for 23 h, and no significant changes in intensity were observed.

The results of EPR measurements did not show any singlet oxygen generation, either after the exposure of the composite samples to blue or green light (Figure 3b). It was shown [59] that functional groups with N quench singlet oxygen. In our previous study [25], XPS analysis showed the presence of NCO and NH groups in BC-ChiD composites, which are responsible for the quenching of singlet oxygen.

### 3.5. *In Vitro* Release of ChiD from the BC-ChiD Composite Hydrogels Exposed to Blue and Green Light

**Figure 4:**
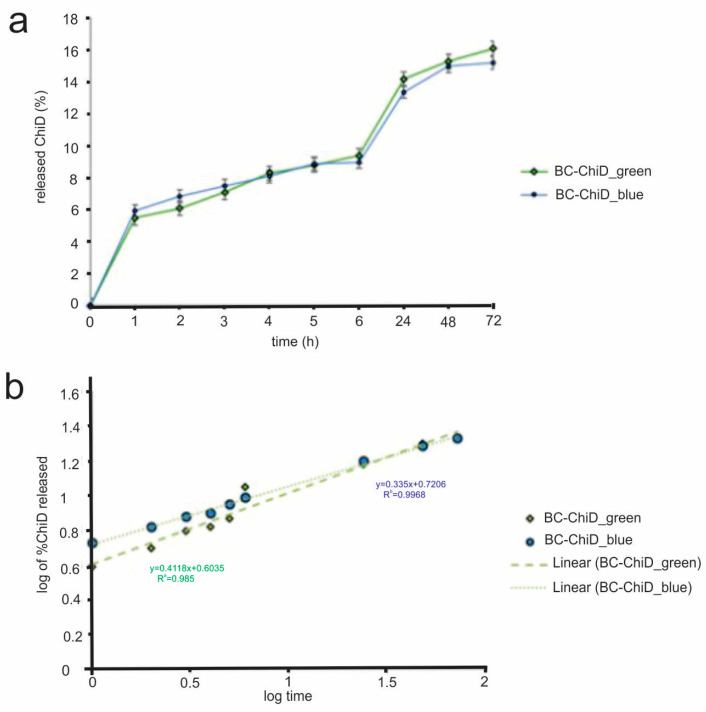
*In vitro* release profile of ChiD from BC-ChiD composites exposed to green and blue light (**a**) and ChiD release data fitted to Korsmeyer–Peppas mathematical kinetic model (**b**).

The release of ChiD from BC-ChiD_green and BC-ChiD_blue composites was observed to be slow and continuous (Figure 4a). At the end of the monitoring period, the total amounts of ChiD released from composites exposed to green and blue light were 16.10 and 15.20, respectively. The mechanism of released ChiD fitted to the Korsmeyer–Peppas model. This kinetic model describes drug release from polymeric systems, yielding the highest R^2^ value. It uses the following equation:

Mt/M∝ = ktn,(5)

where Mt represents the amount of drug released at time t in hours, M∝ is the total amount of drug in dosage form, kt is the kinetic constant, and n is the release exponent related to the mechanism of release. The n values were determined to be 0.335 and 0.412 for BC-ChiD_green and BC-ChiD_blue, respectively, which are both smaller than 0.5 (Figure 4b). This indicates that the release of ChiD from BC-ChiD composites can be characterized as a quasi-Fickian diffusion. The results suggest that the short-term green/blue light illumination of composites does not affect the release of ChiD.

### 3.6. Antibiofilm Activity of BC-ChiD_Blue and BC-ChiD_Green Composite Samples and AFM Imaging of Pathogenic Biofilms after the Application of BC-ChiD_Blue and BC-ChiD_Green Composites

The pathogenic bacteria biofilm reduction after the application of BC-ChiD_blue and BC-ChiD_green composite samples compared to the control samples is shown in Table 2. The reduction in biofilm under blue light was in the range from 48 to 57%, while under green light, it was higher, from 78 to 85%. The most sensitive strain under both blue and green light was *MRSA*.

Changing light conditions alters bacterial growth and metabolism, and it is known that daylight changes the bacterial microbiomes in households [60]. Further light effects are described in different bacteria. *P. aeruginosa* is found to modify pigment and biofilm biomass production under different light conditions [61]. It was shown that *S. aureus*, *E. coli*, and *P. aeruginosa* grow well in a green light environment, while blue light is not as conducive to bacterial growth promotion. This difference results from the presence of endogenous photosensitizing chromophores in pathogenic microbes, revealing that blue light imposes a stress condition on bacteria [62]. Blue light has been shown to cause a reversible decrease in swimming velocity in *Escherichia coli* as a non-phototrophic bacterium. The exact mechanism of this phototactic response is still unknown [63].

The antimicrobial blue light inactivation of biofilms has been showed in many studies [64,65,66,67]. In this study, the biofilms of pathogenic bacteria were exposed to blue and green light for 30 min, a very short time to induce any changes in biofilms themselves but potentially enough to enhance the activity of the new potential antibiofilm composite agent BC-ChiD. Compared to our previous research [34], the composites exposed to ambient light showed a similar antibiofilm effect as BC-ChiD_green against Gram-negative strains (*E. coli*, *K. pneumoniae*, *P. mirabilis* and *P. aeruginosa*). The biofilm reduction in Gram-positive *S. aureus* was higher after the application of BC-ChiD_green than under ambient and blue light. Also, the most sensitive to BC-ChiD_green was *MRSA*, showing higher biofilm reduction compared to *S. aureus*. Since *MRSA* biofilm was not analyzed in our previous study, but it is biologically the *S. aureus* species, it can be presumed that the effect of BC-ChiD under ambient light is probably similar to BC-ChiD_green. All these results show that Gram-positive strains are more sensitive to BC-ChiD_green than Gram-negative. This is in correlation with previous research [15] showing that Gram-negative strains are significantly resistant in the first attempts to photoinactivate bacteria with conventional PSs. Due to the relatively porous layer of peptidoglycan and lipoteichoic acid of Gram-positive strains, ChiD penetrates more easily and adheres to their cell wall.

To date, the reported mechanism of antibiofilm activity of chitosan as a polymer is not as clear as the one against planktonic bacteria. Numerous factors may influence the antibiofilm action: the phase of biofilm formation, molecular weight and deacetylation degree of chitosan, strain specificity (Gram-negative or Gram-positive), etc. [27,68,69]. When seeking to understand the antibiofilm effect of ChiD, it is evident that they have the capability to penetrate deeply into biofilms and exert activity [68], particularly those obtained from low-molecular-weight chitosan [22]. In this study, it was shown that BC-ChiD_green composites had very high antibiofilm potential, which is in correlation with their structure, where ChiDs are distributed mostly on the surface of the composite. Since the charge in ChiD retains a neutral pH, the proposed mechanism of ChiD action is the interaction between the positively charged ChiD and negatively charged bacterial cell membrane, which can lead to the leakage of proteinaceous and other intracellular constituents [22]. This mechanism is the most prevalent proposed antibacterial activity of chitosan [70]. The initial hypothesis of this study, that under blue or green light, photoactive ChiD in composites may produce singlet oxygen and enhance the antibiofilm activity of BC-ChiD composites, is rejected due to EPR results showing no formation of singlet oxygen. Our FTIR results confirmed the presence of amine groups in composites that interact with pathogen membranes and, therefore, lead us to a structure-dependent mechanism of antibiofilm activity. The biofilm mode of life provides microbial communities with ecological advantages, including resistance to mechanical and chemical stresses. However, under blue and green light conditions, BC-ChiD composites succeeded in reducing the biofilm.

**Figure 5:**
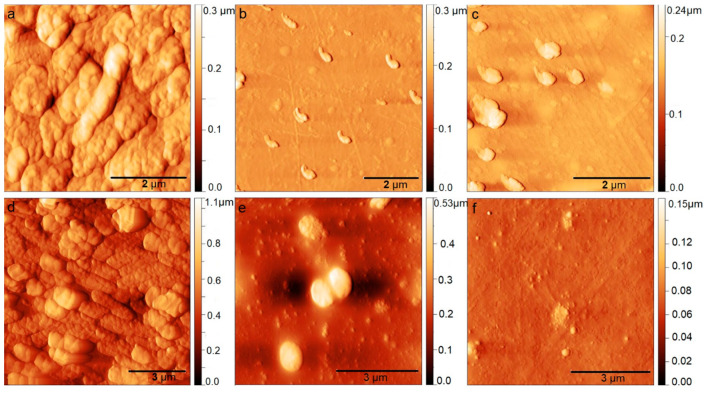
AFM images of *E. coli* and *MRSA* biofilms before the application of BC-ChiD composites ((**a**,**d**), respectively) and after the application of BC-ChiD under blue ((**b**,**e**), respectively) and green ((**c**,**f**), respectively) light.

Figure 5 presents the AFM micrographs of *E. coli* and *MRSA* biofilms before and after the application of BC-ChiD composites irradiated with blue and green light, where a significant reduction in the biofilm was noticed. The biofilm seemed broken into smaller domains, and the average surface roughness (Table 3) significantly reduced after the BC-ChiD_blue and BC-ChiD_green composite application. The surface coverage was also decreased, which is another confirmation of the reduction in the biofilm mass after the treatment with BC-ChiD_blue and BC-ChiD_green composites.

The degradation of biofilm structures in Gram-positive and Gram-negative bacteria in response to external stimuli, such as nanomaterials, antibiotics, heat, and cold plasma, is largely influenced by their cell wall structure. [71]. For irreversible damage to occur in the photodynamic inactivation of bacteria, PSs must accumulate significantly within or on the cytoplasmic membrane of bacteria following irradiation. This process has no effect on *E. coli*, which lacks the uptake of membrane-disorganizing agents, unlike *S. aureus* [72]. The new photoactive composite BC-ChiD releases a sufficient amount of ChiD, which, in a very short time of light irradiation, penetrates and accumulates into the biofilms and shows a high structure-dependent antibiofilm effect.

## 4. Conclusions

In this study, we investigated the antibiofilm activity of a photoactive composite hydrogel comprising bacterial cellulose and low-molecular-weight chitosan dots (BC-ChiD_blue and BC-ChiD_green) under blue and green light exposure, respectively. Our comprehensive analysis using SEM, AFM, FTIR, XRD, porosity measurements, *in vitro* release, and EPR spectroscopy revealed significant structural changes in the BC-ChiD composites following exposure to blue and green light. Notably, BC-ChiD_green exhibited a significantly higher antibiofilm effect (78–85%) compared to BC-ChiD_blue (48–57%), with methicillin-resistant *Staphylococcus aureus* (*MRSA*) being the most sensitive strain. The total amount of released ChiD was up to 16.10%, and the short-term green/blue light illumination of composites did not affect the release of ChiD.

The absence of singlet oxygen formation suggests a structure-dependent mechanism of antibiofilm activity, potentially involving the interaction between amine groups of ChiDs and negatively charged bacterial cell membranes, leading to bacterial destruction. These findings underscore the potential utility of BC-ChiD_blue and BC-ChiD_green in eradicating pathogenic biofilms on open surfaces in biomedical applications, including wound healing and the prevention of foodborne and nosocomial infections.

Moving forward, future research will focus on elucidating the specific molecular mechanisms underlying the antibiofilm activity of BC-ChiD composites and further optimizing their formulation for enhanced efficacy. Additionally, investigating the long-term stability and biocompatibility of these composites in vivo could provide valuable insights into their potential clinical applications. Furthermore, exploring alternative light sources and dosing regimens to maximize antibiofilm activity while minimizing potential side effects could be beneficial for the development of more effective photodynamic therapy strategies against biofilm-related infections.

## Figures and Tables

**Table 1:** Crystallite size and crystallinity index comparison among BC-ChiD_green, BC-ChiD_blue, and BC-ChiD_control samples.

Sample	Crystallite Size (nm)	Crystallinity Index (%)
BC-ChiD_blue	4.7	30.7
BC-ChiD_green	4.3	32
BC-ChiD_control [25]	2.2	74

**Table 2:** Microtitre biofilm reduction assay. Pathogenic bacteria biofilm reduction (presented in %) after the application of BC-ChiD composites exposed to blue and green light. The values 0.2 and 2 refer to the concentration of ChiD solutions used to prepare the composites.

Bacteria	BC-ChiD_Blue		BC-ChiD_Green	
	0.2	2	0.2	2
*S. aureus*	48	52	81	80
*MRSA*	57	56	80	85
*E. coli*	51	51	78	79
*K. pneumoniae*	47	57	78	79
*P. mirabilis*	54	45	80	70
*P. aeruginisa*	54	52	80	70

**Table 3:** Average surface roughness (RMS) and surface area of pathogenic bacteria biofilms (presented in nm and µm^2^, respectively) after the application of BC-ChiD_blue and BC-ChiD_green.

Bacteria	BC-ChiD_Control	BC-ChiD_Blue	BC-ChiD_Green
	Average surface roughness—RMS (nm)		
*E. coli*	93.46 ± 22.66	16.66 ± 4.46	71.6 ± 16.29
*MRSA*	138.33 ± 24.96	58.7 ± 8.93	14.43 ± 3.47
	Surface area [µm^2^]		
*E. coli*	144.07 ± 11.95	107.32 ± 1.97	110.41 ± 3.92
*MRSA*	162.06 ± 6.35	113.08 ± 2.18	104.27 ± 0.68

## Data Availability

The data presented in this study are available on request from the corresponding author.
